# Hellinger distance-based stable sparse feature selection for high-dimensional class-imbalanced data

**DOI:** 10.1186/s12859-020-3411-3

**Published:** 2020-03-23

**Authors:** Guang-Hui Fu, Yuan-Jiao Wu, Min-Jie Zong, Jianxin Pan

**Affiliations:** 1 School of Science, Kunming University of Science and Technology, Kunming, 650500 People’s Republic of China; 2 School of Mathematics, The University of Manchester, Manchester, M13 9PL UK

**Keywords:** Hellinger distance, Class-imbalance learning, Feature selection, Sparse regularization

## Abstract

**Background:**

Feature selection in class-imbalance learning has gained increasing attention in recent years due to the massive growth of high-dimensional class-imbalanced data across many scientific fields. In addition to reducing model complexity and discovering key biomarkers, feature selection is also an effective method of combating overlapping which may arise in such data and become a crucial aspect for determining classification performance. However, ordinary feature selection techniques for classification can not be simply used for addressing class-imbalanced data without any adjustment. Thus, more efficient feature selection technique must be developed for complicated class-imbalanced data, especially in the context of high-dimensionality.

**Results:**

We proposed an algorithm called sssHD to achieve stable sparse feature selection applied it to complicated class-imbalanced data. sssHD is based on the Hellinger distance (HD) coupled with sparse regularization techniques. We stated that Hellinger distance is not only class-insensitive but also translation-invariant. Simulation result indicates that HD-based selection algorithm is effective in recognizing key features and control false discoveries for class-imbalance learning. Five gene expression datasets are also employed to test the performance of the sssHD algorithm, and a comparison with several existing selection procedures is performed. The result shows that sssHD is highly competitive in terms of five assessment metrics. In addition, sssHD presents limited differences between performing and not performing re-balance preprocessing.

**Conclusions:**

sssHD is a practical feature selection method for high-dimensional class-imbalanced data, which is simple and can be an alternative for performing feature selection in class-imbalanced data. sssHD can be easily extended by connecting it with different re-balance preprocessing, different sparse regularization structures as well as different classifiers. As such, the algorithm is extremely general and has a wide range of applicability.

## Background

Feature selection has recently gained considerable attention in class-imbalance learning due to the high-dimensionality of class-imbalanced data across many scientific disciplines [1–3]. To date, a variety of feature selection methods have been proposed to address high-dimensional data. However, only a small number of them are technically designed to handle the problem of class distribution under a class-imbalance setting [4–7]. Thus, performing feature selection from class-imbalanced data remains a challenging task due to the inherent complex characteristics of such data, and a new understanding or principle is required to efficiently transform vast amounts of raw data into information and knowledge representation [8].

Feature selection can simplify data by eliminating uninformative predictors as well as selecting the key biomarkers for a certain task. In addition, feature selection is an effective strategy to alleviate the overlap caused by the interaction of high-dimensionality and class-imbalance [5, 9, 10]. In fact, standard classifiers can still produce good discrimination for some highly class-imbalanced data sets if the data (or two class distributions) at hand can be well separated, regardless of the class-imbalanced ratio and the lack of data. However, overlapping (non-separability) usually occurs in the settings of high-dimensionality and class-imbalance. An instance from class *C* belongs to an overlapping region if out of its *k* nearest neighbors, more than *h* (such as *h*=[*k*/2]) belong to a class other than *C*. Overlapping happens when a similar amount of training data for each class is mixed in the overlapping region. When overlapping arises, it is very difficult or impossible to separate a class from others. Some findings have shown that overlap can play an even larger role in determining classifier performance than class-imbalance [11]. As far as high-dimensional and class-imbalanced data is concerned, it is worthwhile to investigate the way to alleviate the overlap effectively with feature selection.

There are three categories of feature selection in the context of classification, depending on how these feature selection searches combine with the construction of the classification model: filtering, wrapping and embedding [12]. The filtering method assesses the relevance of features by looking only at the intrinsic properties of the data, and selects high-ranking features based on a statistical or information measure, such as information gain and gain ratio [13]. There are two drawbacks of filter-based selection: first, the filtering selection is independent of the classifier, which may lead to reduced classification accuracy with a certain kind of classifier; and second, it ignores the dependencies among features. Such dependency information should be considered in performing variable selection as strongly related features are often similar and should be aggregated. The related features may play an important role in performing feature selection, especially in high-dimensional settings. The wrapping method, such as a genetic algorithm [14], wraps a search algorithm around the classification model to search the space of all feature subsets. However, an obvious drawback of the wrapping method is that it is computationally intensive, as the number of subsets from the feature space grows exponentially as the number of features increases. The embedding method screens out key features while considering the construction of a classifier, such as LASSO-based feature selection and classification [15]. It is integrated in the modelling process and is classifier-dependent [12].

In class-imbalance scenarios, filtering and wrapping feature selection methods were the most frequently built and were used to solve real-world problems, such as disease diagnosis, textual sentiment analysis, and fraud detection [16]. Dozens of metrics and their variants are employed for building filter-based feature selection algorithms in many research studies [7, 17–19], such as odds-ratio, chi-squared, Relief, ReliefF, information gain, gain ratio, Gini index, *F*−*measure*, *G*−*mean*, signal-to-noise ratio, and area under receiver operating characteristics (ROC) graph. To effectively reduce the computation cost, wrapping feature selection techniques usually utilize ad hoc search strategies, such as a heuristic search [20, 21] and stochastic search [22]. To the best of our knowledge, embedding feature selection methods are less investigated than filtering and wrapping methods. One of the related studies is from the reference [23], which considers sparse logistic regression with stable selection in handling Alzheimer’s disease neuroimaging initiative dataset and stated that it achieved competitive performance compared with several filter-based selection methods based on their experimental results.

**Fig. 1:**
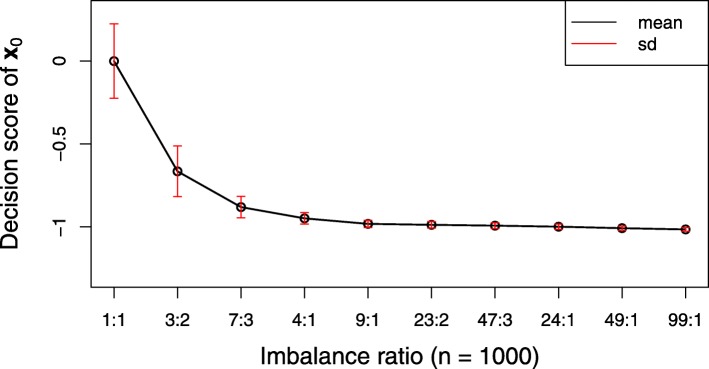
Decision score of the SVM classifier varies greatly as the class-imbalanced ratio changes. In this simulation, the *X*-matrix (1000×3) is randomly produced then fixed while the class label *y*=±1 is randomly changed at each iteration under the fixed class-imbalanced ratio. The prediction point is set be **x**_0_=(−5,9,−1)^*T*^. The black line and the red vertical short segments are, respectively, the mean and standard deviation of prediction decision score with the class-imbalanced ratio changing from 1 to 99 based on 2000 iterations

Considering the drawbacks of filtering and wrapping algorithms, in this study, we focus on the embedding-based selection algorithm by bringing in sparse regularization [24]. Common embedding feature selection methods for classification can not be used simply for addressing class-imbalanced data without any adjustment because of the following issues:

(a) These standard selection algorithms are generally based on the assumption of balanced class distributions, and the selection results are affected due to the class-imbalanced ratio between classes and consequently produce highly biased classification prediction towards the majority class.

(b) The classifier’s continuous output, to some extent, may shift due to the domination of the majority class [25, 26]. Figure 1 exhibits such shifting based on a support vector machine (SVM) classifier. It can be seen that the decision score varies greatly as the class-imbalanced ratio changes. When the threshold in decision function still keeps the default value (e.g., 0 in SVM and 0.5 in logistic regression), the decision boundary (or the separating hyperplane) must be shifted towards the minority. From the view of geometry, the separating hyperplane would be shifted towards the minority class due to the domination of the majority class. One of the attempts for handling this question is threshold adjustment[25, 27–29] via moving the decision threshold towards the majority examples so that the minority class examples become harder to misclassify. However, as was pointed out in the references [25, 26], it is difficult to decide how far the separating hyperplane should be moved towards the majority class, and such adjustment may over-correct the decision boundary towards the majority class, which leads to increasing error on the majority class.

(c) Common feature selection measurements are not suitable in class-imbalance learning. Traditionally, feature selection techniques were developed to maximize the total classification accuracy of a classifier. As is well-known, the majority class is more influential than the minority class in performing feature selection.

In this study, the Hellinger distance is employed as an assessment measurement on the assumption of binormal distributions to combat class-imbalance and output-shifting in class-imbalance learning.

## Methods

### Hellinger distance under binormal assumption

The Hellinger distance is a measure of the distributional divergence [30]. Let *P* and *Q* be two probability measures that are absolutely continuous with respect to a third probability measure *λ*. The square of the Hellinger distance can be defined as follows:

1 $$  D_{H}^{2}(P,Q) = \int\left(\sqrt{\frac{dP}{d\lambda}}-\sqrt{\frac{dQ}{d\lambda}}\right)^{2}d\lambda  $$

Here, *λ* is set be the Lebesgue measure, so that $\frac {dP}{d\lambda }$ and $\frac {dQ}{d\lambda }$ are two probability density functions. Based on the binormal assumption, *P* and *Q* are two normal distributions, and

2 $$ \left\{\begin{array}{l} \frac{dP}{d\lambda} = f_{1}(x) \sim N\left(\mu_{1}, \sigma_{1}^{2}\right),\\ \\ \frac{dQ}{d\lambda} = f_{0}(x) \sim N\left(\mu_{0}, \sigma_{0}^{2}\right) \end{array} \right.  $$

Thus Eq. (1) can be rewritten as

3 $$\begin{array}{@{}rcl@{}}  D_{H}^{2}(P,Q)&=&\int\left(\sqrt{f_{1}(x)}-\sqrt{f_{0}(x)}\right)^{2}dx\\ &=&2 - 2\int\sqrt{f_{1}(x)f_{0}(x)}dx\\ &=&2-2\sqrt{\frac{2\sigma_{1}\sigma_{0}}{\sigma_{1}^{2} + \sigma_{0}^{2}}}Exp\left\{-\frac{(\mu_{1}-\mu_{0})^{2}}{4\left(\sigma_{1}^{2} + \sigma_{0}^{2}\right)}\right\} \end{array} $$

In practice, the parameters *μ*_1_, $\sigma _{1}^{2}$, *μ*_0_, and $\sigma _{0}^{2}$ can be replaced by the corresponding sample statistics $\bar {X}_{1}$, $S_{1}^{2}$, $\bar {X}_{0}$, and $S_{0}^{2}$, respectively.

Without a loss of generality, let **y**=(*y*_1_,*y*_2_,⋯,*y*_*n*_)^*T*^ be binary categorical response and *y*_*i*_=1 if it belongs to the minority class (positive) and *y*_*i*_=−1 if it belongs to the majority class (negative); let ***x***_*j*_ be the *j*^*th*^ feature (*j*=1,2,⋯,*p*) and **X**=(***x***_1_,***x***_2_,⋯,***x***_*p*_); and let ***β***=(*β*_1_,*β*_2_,⋯,*β*_*p*_)^*T*^ be the vector of estimate coefficients. The normality assumption here is on a linear combination of the predictor matrix **X** rather than each single feature, namely

4 $$ P = \textbf{X}\boldsymbol{\beta}\Big|(\textbf{y} = 1)=\beta_{1}{\boldsymbol{x}}_{1} + \beta_{2}{\boldsymbol{x}}_{2} + \cdots + \beta_{p}{\boldsymbol{x}}_{p}\Big|(\textbf{y} = 1),  $$

5 $$ Q = \textbf{X}\boldsymbol{\beta}\Big|(\textbf{y} = -1)=\beta_{1}{\boldsymbol{x}}_{1} + \beta_{2}{\boldsymbol{x}}_{2} + \cdots + \beta_{p}{\boldsymbol{x}}_{p}\Big|(\textbf{y} = -1)  $$

Obviously, the binormal assumption on a linear combination would be more likely to hold than a single variable, particularly for moderate to large size of features *p* according to the central limit theorem (CLT).

**Definition** 1. A quantity is called skew-insensitive if it is not influenced by the class priors.

**Definition** 2. Let *t*_*i*_(*i*=1,2,⋯,*n*) be the original score of each observation and *c* be a constant (*c*≠0). A quantity is called translation-invariant if it remains unchanged when each score moves to *t*_*i*_+*c*,(*i*=1,2,⋯,*n*).

**Property** 1. The Hellinger distance is skew-insensitive under binormal assumption.

**Fig. 2:**
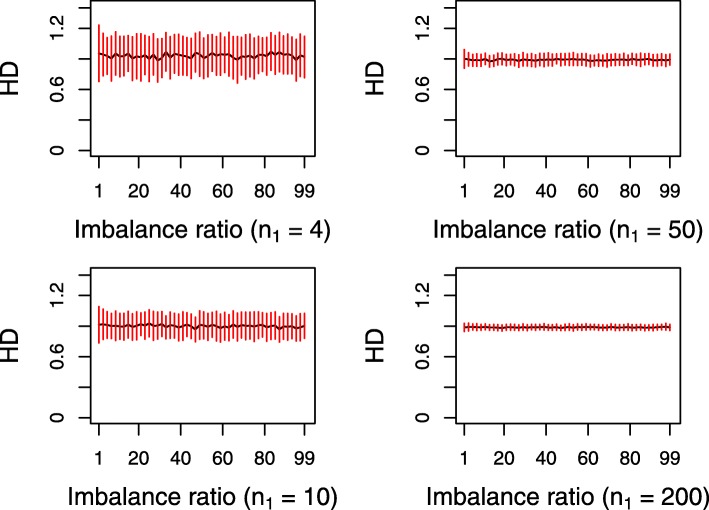
Skew-insensitivity of the Hellinger distance on simulated normal distributions with four scenarios corresponding to different sizes of the minority. The black line and red vertical short segments are, respectively, the mean and standard deviation of the Hellinger distance with the ratio changing from 1 to 99 based on 100 iterations

Equation (3) shows that the computation of the Hellinger distance is not influenced by the class-imbalanced ratio. It is just relevant with the expectations *μ*_0_, *μ*_1_ as well as variances $\sigma _{0}^{2}$, $\sigma _{1}^{2}$ of *P* and *Q*. The law of large numbers tells us that these four numerical characteristics are approached by their corresponding sample statistics if the sample size is large enough. They are independent of the class-imbalanced ratio. An example is given in Fig. 2 to exhibit this skew-insensitivity by means of calculating the magnitude of the Hellinger distance on two normal distributions. It can be seen from Fig. 2 that the value of the Hellinger distance stays consistent when the class-imbalanced ratio changes from 1 to 99, and such consistency tends to become increasingly true as the sample size increases. Namely, the magnitude of the Hellinger distance is not influenced by the class-imbalanced ratio. In fact, such skew-insensitivity has also been shown in the reference [31] in terms of comparing isometrics and giving a synthetic example.

**Property** 2. Hellinger distance is translation-invariant under binormal assumption.

Considering that two variances $\sigma _{0}^{2}$ and $\sigma _{1}^{2}$ as well as the difference *μ*_1_−*μ*_0_ keep invariant as each score moves, the Hellinger distance will stay the same according to Eq. (3).

As mentioned above, Hellinger distance essentially captures the divergence between the feature value distributions of different classes and is not influenced by the class ratios under binormal assumption. This is the motivation why Hellinger distance is utilized for class-imbalanced data in this study. In addition, its translation-invariant is very useful to combat the output-shifting arisen when a standard classifier is used to distinguish class-imbalanced data. Unlike the usage of Hellinger distance in the previous work [5, 31], where the feature attributes should be discrete or to discretize the continuous features for the calculation of Hellinger distance, Hellinger distance in this study can be calculated directly based on continuous variables without discretization.

### Hellinger distance-based stable sparse selection (sssHD) and its algorithm

Considering the above questions from class-imbalance learning and being motivated by the properties of the Hellinger distance, we proposed a Hellinger distance-based stable sparse selection (sssHD) approach to perform feature selection when the category data is class-imbalanced. An ordinary classifier generally can not perform feature selection automatically, but a kind of sparse penalty coupled with the Hellinger distance metric can be embedded into the classifier to achieve such a task. For convenience, a linear SVM classifier is employed as an example to establish our sssHD algorithm.

SVM [32–35] has shown promising capability in solving many classification problems. It performs two-classification task by constructing a hyperplane in the multidimensional space to differentiate two classes with a maximal margin. Let **x**_*i*_=(*x*_*i*1_,*x*_*i*2_,⋯,*x*_*ip*_)^*T*^ be the *i*^*th*^ instance and its class label *y*_*i*_=1 or − 1 (*i*=1,2,⋯,*n*). The decision function of SVM can be expressed as follows:

6 $$  f(\textbf{x}_{i})=\bigg\{\begin{array}{lll} 1,\hspace{5mm}\boldsymbol{\beta}^{T}\textbf{x}_{i}+\beta_{0} > 0,\\ -1,\hspace{2mm}\boldsymbol{\beta}^{T}\textbf{x}_{i}+\beta_{0} \leq 0\\ \end{array}  $$

where *β*_0_ is the constant coefficient, and ***β***^*T*^**x**_*i*_+*β*_0_ is called the decision score in this study. The soft margin support vector classifier can be estimated by solving the following quadratic optimization problem:

7 $$  \min \hspace{6mm} \frac{1}{2}\|\boldsymbol{\beta}\|^{2}+C\sum_{i=1}^{n}\xi_{i},  $$

8 $$  s.t.\hspace{6mm}\bigg\{\begin{array}{ll} y_{i}(\boldsymbol{\beta}^{T}\textbf{x}_{i}+\beta_{0})\geq 1-\xi_{i},\\ \xi_{i}\geq0,\hspace{4mm}(i=1,2,\cdots,n),\\ \end{array}  $$

where ***ξ***=(*ξ*_1_,*ξ*_2_,⋯,*ξ*_*n*_)^*T*^ are slack variables which are associated with the misclassified individuals. Formula (7) with constraint (8) can be rewritten as

9 $$  \min \hspace{3mm} Loss + \lambda\|\boldsymbol{\beta}\|^{2}_{2},  $$

where $Loss = \sum _{i=1}^{n}max(0, 1- y_{i}(\boldsymbol {\beta }^{T}\textbf {x}_{i}+\beta _{0}))$ is hinge loss, and $\|\boldsymbol {\beta }\|_{2}^{2}=\sum _{j=1}^{p}\beta _{j}^{2}$ is the ridge penalty. The ridge penalty shrinks the estimation coefficients towards zero and, hence, possibly improves the model’s prediction accuracy; however, it can not perform feature selection automatically. Therefore, the ridge penalty should be replaced by a sparse regularization penalty to induce the sparsity for achieving feature selection. Sparse selection is a very popular technique to perform variable selection for high-dimensional data [24, 36–39]. Taking elastic-net [38] as an example, it is defined as follows:

10 $$  C_{\alpha}(\boldsymbol{\beta})=\frac{1}{2}(1-\alpha)\|\boldsymbol{\beta}\|_{2}^{2}+\alpha\|\boldsymbol{\beta}\|_{1},  $$

where $\|\boldsymbol {\beta }\|_{1}=\sum _{j=1}^{p}|\beta _{j}|$ is LASSO penalty[24], and *α*∈[0,1]. Actually, the elastic-net penalty is a combination of ridge and LASSO penalties, which is particularly useful and effective for feature selection, especially when the data is strongly correlated and high-dimensional. Sparse support vector machine with elastic-net penalty can be expressed as

11 $$  \min \hspace{3mm} Loss + \lambda C_{\alpha}(\boldsymbol{\beta}),  $$

where *λ* is the tuning parameter that controls the tradeoff between loss and penalty.

The optimal estimation $(\hat {\boldsymbol {\beta }}, \hat {\beta _{0}})$ in objective (11) is the function of *λ* and *α*. Consequently, the decision score $\hat {\textbf {t}} =(\hat {t}_{1},\hat {t}_{2},\cdots,\hat {t}_{n})^{T}= \textbf {X}\hat {\boldsymbol {\beta }} + \hat {\beta _{0}}$ is also influenced by *λ* and *α*. Denoted $\hat {\textbf {t}}$ by $\hat {\textbf {t}}(\lambda, \alpha) = (\hat {\textbf {t}}_{0}(\lambda,\alpha), \hat {\textbf {t}}_{1}(\lambda,\alpha))^{T}$, where $\hat {\textbf {t}}_{0}(\lambda,\alpha) =\{\hat {t}_{i}|y_{i} = -1\}$ and $\hat {\textbf {t}}_{1}(\lambda,\alpha) =\{\hat {t}_{i}|y_{i} = 1\}$, the objective of sparse selection with Hellinger distance can be defined as

12 $$  \hat{\boldsymbol{\beta}} = \max \hspace{3mm} D_{H}\left(\hat{\textbf{t}}_{0}(\lambda,\alpha), \hat{\textbf{t}}_{1}(\lambda,\alpha)\right)  $$

A potential question of sparse feature selection is its instability caused by the variation from the training data [40, 41]. Class-imbalance is going to exacerbate this drawback. A decent strategy to overcome such disadvantage is to combine sparse selection with subsampling. Meinshausen et al. [40] pointed out that such marriage yields finite sample family-wise error control and significantly improves selection methods. In this study, objective (12) is conducted many times with subsampling to achieve stable selection. Denoted $\hat {\boldsymbol {\beta }}$ from objective (12) by $\hat {\boldsymbol {\beta }}^{(k)}$ in the *k*^*th*^ subsampling (*k*=1,2,⋯,*K*). The importance of the features is measured by the inclusion frequency, which is denoted by **f**=(*f*_1_,*f*_2_,⋯,*f*_*p*_)^*T*^, and is defined as follows:

13 $$  f_{j} = \frac{1}{K}\sum_{k=1}^{K}g\left(\hat{\beta}^{(k)}_{j}\right), \hspace{5mm}j = 1, 2, \cdots, p,  $$

where $g\left (\hat {\beta }^{(k)}_{j}\right) = 1$ if $\hat {\beta }^{(k)}_{j} \neq 0$, otherwise $g\left (\hat {\beta }^{(k)}_{j}\right) = 0$. All the features are ranked with their inclusion frequencies, and the feature with maximal inclusion frequency is the most important. More details of the sssHD algorithm is given in Algorithm 1. The ratios of subsampling from the majority (*r*_0_) and minority (*r*_1_) are set to be equal in this study to keep the class-imbalance ratio of the subset the same as the original data. sssHD is extremely general and can be easily extended; for example, sparse SVM can be placed by sparse logistic regression [42] or Fisher linear discriminant [43]; re-balance methods such as over-sampling [44] or under-sampling [45] could be connected if necessary; sparse regularization (Eq. (10)) also has many alternatives, such as SCAD [36], adaptive LASSO [39], group LASSO [46], and group bridge penalty [47].


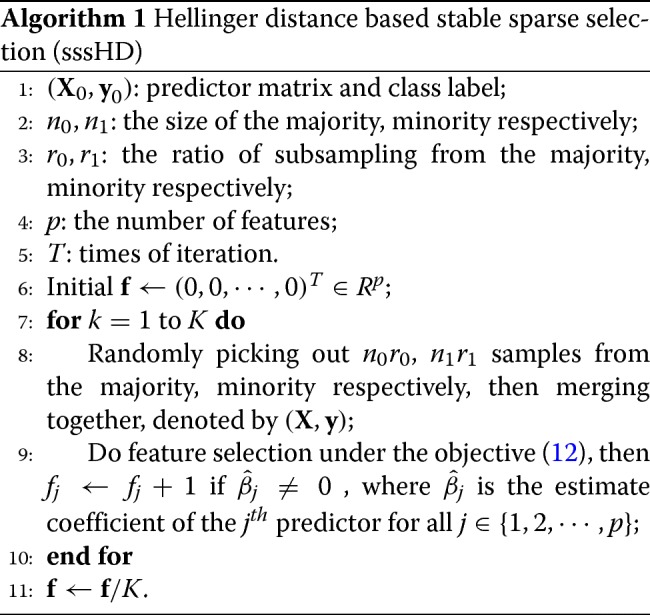


### Assessment metrics and experimental methods

In class-imbalance learning, the majority and the minority are generally called as negative and positive, respectively. A binary classifier predicts all the instances as either positive or negative. Thus, it produces four types of outcome: true positive (*TP*), true negative (*TN*), false positive (*FP*) and false negative (*FN*). Several metrics can be defined according to these outcomes, such as

$$\begin{array}{*{20}l} &TPR = recall = \frac{TP}{TP + FN} = \frac{TP}{n_1};\\ &TNR = \frac{TN}{TN + FP} = \frac{TN}{n_{0}};\\ &FPR = \frac{FP}{FP + TN} = \frac{FP}{n_{0}};\\ &precision = \frac{TP}{TP + FP};\\ &G-mean = \sqrt{TPR \times TNR};\\ &F-measure = \frac{2(precision \times recall)}{precision + recall} \end{array} $$

As shown in above, *precision* is the proportion of true positives among the positive predictions; *recall* (*TPR*) measures the proportion of positives that are correctly identified; *G*−*mean* is the geometric mean of *TPR* and *TNR*, which measures the accuracy on both the majority class and the minority class; *F*−*measure* is a kind of combinations of *precision* and *recall*, and is high when both of them are high.

ROC curve can be created by plotting *TPR* on the y-axis against *FPR* on the x-axis at various threshold settings. Let *T*,*T*_1_ and *T*_0_ denote respectively the continuous outputs for total, positive and negative examples by the binary classifier (such as SVM); *T* is the mixture of *T*_1_ and *T*_0_. Larger output values are associated with positive examples. So for a given threshold *c* (−*∞*<*c*<+*∞*), an example is predicted positive if its value is greater than *c*. Thus,

$$TPR(c) = P(T_{1}>c)=P(T>c|y=1),$$

$$FPR(c) = P(T_{0}>c)=P(T>c|y=0)$$

A ROC curve may be defined as the set of points:

$$ROC(\cdot) = \left\{\left(TPR(c), FPR(c)\right) \Big|-\infty< c< +\infty)\right\}$$

The area under ROC (*AUCROC*) is calculated here by using trapezoidal rule [48], where the point with the minimal value at this *FPR* is linked to the point with the maximal value at the next *FPR* value when there is more than one value at the same *FPR* value. Let *F*_1_,*F*_2_,⋯,*F*_*K*_ be all the different *FPR* values satisfying *F*_1_<*F*_2_<⋯<*F*_*K*_, and $T_{k}^{max}$ and $T_{k}^{min}$ are the maximal and minimal *TPR* values corresponding to *F*_*k*_ (*k*=1,2,⋯,*K*), respectively. The empirical AUCROC with the lower trapezoidal rule is

14 $$  AUCROC = \sum_{k=1}^{K-1}\frac{1}{2}\left(T_{k}^{min} + T_{k+1}^{max}\right)(F_{k+1}-F_{k})  $$

In this study, *TPR*, *G*−*mean*, *F*−*measure*, *AUCROC* and *precision* are employed as assessment metrics to perform real data experiments. Cross validation is performed for each real data in computing these measures. In order to keep the invariant of the imbalance ratio in each fold, stratified sampling is utilized. Namely each fold contains the same size of negative (and positive) instances and their class-imbalance ratios are equal to the ratio of original data set.

To evaluate sssHD algorithm with the above real data sets, we compare it with other four filter-based feature selection methods: Fisher score [49], Relief [50], area under receiver operating characteristic (AUCROCfilter) [51] and area under precision-recall curve (AUCPRCfilter) [52].

**Fisher score**: the Fisher score could strongly depend on the directions of the spread of the data by calculating the difference of each feature’s mean values in two classes:

15 $$  F_{j} = \frac{\left |\mu_{1j} - \mu_{0j}\right |}{\sigma_{1j}^{2} + \sigma_{0j}^{2}}, \hspace{5mm}j = 1, 2, \cdots, p,  $$

where *μ*_0*j*_, *μ*_1*j*_, $\sigma _{0j}^{2}$, and $\sigma _{1j}^{2}$ are respectively the mean and the variance of the *j*^*th*^ predictor of the majority and the minority. Attributes with a higher score are more important for separating the two classes. Fisher score has been utilized successfully in many classification issues [53, 54].

**Relief**: the Relief is a randomized algorithm that attempts to give each predictor a weight indicating its level of relevance to the target. In each iteration, Relief first needs to search two nearest neighbors for any selected example point (**x**_*i*_): one from the same class (**nearhit**_*i*_), and one from the other class (**nearmiss**_*i*_); then, if Euclidean distance is employed, the weight vector **w**=(*w*_1_,*w*_2_,⋯,*w*_*p*_)^*T*^ is updated so that

16 $$ {\begin{aligned} w_{j} \longleftarrow w_{j} - (x_{ij} - nearhit_{ij})^{2} &+ (x_{ij}- nearmiss_{ij})^{2},\\&\quad \hspace{4mm}j = 1, 2, \cdots, p, \end{aligned}}  $$

where *x*_*ij*_, *nearhit*_*ij*_ and *nearmiss*_*ij*_ correspond to the *j*^*th*^ component of **x**_*i*_, **nearhit**_*i*_ and **nearmiss**_*i*_, respectively. Attributes with a larger weight are more relevant with the response. The Relief method can be applied to a variety of complicated situations and now has several generalizations, such as ReliefF [55].

**AUCROCfilter**: as stated above, ROC can be used as a metric to evaluate the final model. In addition, ROC and its area could be used as a filter feature selection method when just considering a single predictor each time. To obtain the predicted class labels, ROC should be combined with a classifier. Obviously, an attribute with larger area under the curve is more important for classifying the target. An ROC-based filter feature selection strategy has been used for high-dimensional class-imbalanced data [51].

**AUCPRCfilter**: as an alternative of ROC, the precision-recall curve (PRC) has gained increased attention recently in class-imbalance learning [56]. The PRC curve is created by plotting the recall on the x-axis against precision on the y-axis at various threshold settings. The area under PRC (AUCPRC) can be seen as the average of the precision weighted by the probability of a given threshold and is utilized to define how a classifier performs over the whole space. Similarly, AUCPRC coupled with a classifier can be used individually as a filter-based feature selection method for each attribute. Attributes with larger AUCPRC are more significant for separating classes.

## Results and discussion

### Simulation study

In this section, we test our HD-based method with simulation data in a range of settings, comparing it to another two embedded feature selection methods: classification accuracy (ACC)-based and ROC-based sparse selection techniques.

#### Simulation data

The **X**-matrix corresponding to two classes are separately generated via multivariate normal distributions. Namely, **X**|(*y*=0)∼*N*_*p*_(***μ***_0_,***Σ***) and **X**|(*y*=1)∼*N*_*p*_(***μ***_1_,***Σ***). Here ***μ***_0_≠***μ***_1_, namely, the predictors in two classes have the same covariance but different mean value. The first ten variables are set to be key features and the rest are null variables. The difference between ***μ***_1_ and ***μ***_0_ is zero for null features, but two for key features. ***Σ*** is a blocked correlation matrix, with off-diagonal elements of *ρ*^|*i*−*j*|^ for all *i*,*j*=1,2,⋯,10 (key features), and *ρ*^|*i*−*j*|^ for all *i*,*j*=11,12,⋯,*p* (irrelevant features). Between-block correlation is zero. The size of total samples is fixed (namely *n*_0_+*n*_1_=960), whereas the number of predictors *p* is set be, on one hand, 100 to evaluate an over-determined case (*n*>*p*), and on the other hand, 2000 to assess an under-determined situation (*n*<*p*). The majority to the minority ratio here is set be 1 : 1, 3 : 1, 9 : 1 and 15 : 1, respectively. Low (*ρ*=0), moderate (*ρ*=0.4) and high (*ρ*=0.92) correlation structures are simulated under the above settings. The R code for generating this simulation data can be found in the supplementary information.

#### Computation and results

SVM with sparse penalty (10) is employed here to perform feature selection. Three measurements, namely ACC, ROC and HD, are utilized to search the optimal parameters in sparse SVM. To be fair, subsampling is not involved in computation with HD and all the model parameters are set to be same for three algorithms. The mean of the number of correctly (*C*) and incorrectly (*IC*) selected predictors are calculated based on 500 trials and the results are reported in Table 1. *C* corresponds to the number of selected variable from 10 key features. It can be seen that most of key features are correctly selected by using HD, especially when the correlation structures among predictors are not too high. In addition, HD performs best compared with ACC and ROC in terms of *C* in most situations, which means that the statistical power of HD-based technique is extremely competitive in comparison with other two assessments. *IC* is actually the number of selected features from the null variables. Table 1 shows that *IC* from HD is quite low. Considering a large number of null features, the false discoveries from HD are much less than that from both ACC and ROC in almost all the cases. Therefore HD-based selection is suitable to recognize key features and control the false discoveries.

**Table 1:** The feature selection result on simulation data in which 10 key biomakers are included. Mean reported based on 500 replications

				*C*				*IC*		
*p*/*r*	Ratio	*n*_0_	*n*_1_	ACC	ROC	HD		ACC	ROC	HD
A: *p*=100,*r*=0	1 : 1	480	480	8.43	2.76	9.93		0.10	0.09	0.00
	3 : 1	720	240	10.00	2.95	10.00		3.79	0.77	0.04
	9 : 1	864	96	10.00	6.28	10.00		19.64	1.33	0.42
	15 : 1	900	60	10.00	7.95	10.00		60.36	0.85	1.77
B: *p*=100,*r*=0.4^|*i*−*j*|^	1 : 1	480	480	9.96	9.86	9.96		0.25	0.13	0.01
	3 : 1	720	240	10.00	9.96	9.99		6.67	0.90	0.14
	9 : 1	864	96	10.00	9.98	10.00		57.64	3.09	1.14
	15 : 1	900	60	3.80	9.91	10.00		31.01	3.23	1.85
C: *p*=100,*r*=0.92^|*i*−*j*|^	1 : 1	480	480	9.98	10.00	10.00		0.51	0.63	0.57
	3 : 1	720	240	10.00	9.84	9.90		1.58	0.59	0.47
	9 : 1	864	96	0.69	8.01	8.01		5.87	0.67	0.59
	15 : 1	900	60	0.00	6.85	6.85		0.00	0.84	0.71
D: *p*=2000,*r*=0	1 : 1	480	480	7.89	2.79	9.94		0.10	0.04	0.00
	3 : 1	720	240	10.00	3.20	10.00		5.08	1.54	0.03
	9 : 1	864	96	10.00	5.83	10.00		98.24	1.83	0.20
	15 : 1	900	60	10.00	7.38	10.00		404.96	5.94	2.13
E: *p*=2000,*r*=0.4^|*i*−*j*|^	1 : 1	480	480	9.97	9.91	10.00		0.28	0.18	0.01
	3 : 1	720	240	10.00	9.91	10.00		13.06	1.06	0.16
	9 : 1	864	96	10.00	9.95	10.00		293.50	4.30	0.57
	15 : 1	900	60	10.00	9.93	9.98		711.05	6.34	2.71
F: *p*=2000,*r*=0.92^|*i*−*j*|^	1 : 1	480	480	9.95	10.00	10.00		0.76	0.54	0.53
	3 : 1	720	240	10.00	9.73	9.72		4.05	0.28	0.25
	9 : 1	864	96	3.15	7.95	7.95		161.82	0.68	0.56
	15 : 1	900	60	1.07	6.62	6.62		107.61	0.69	0.58

**Fig. 3:**
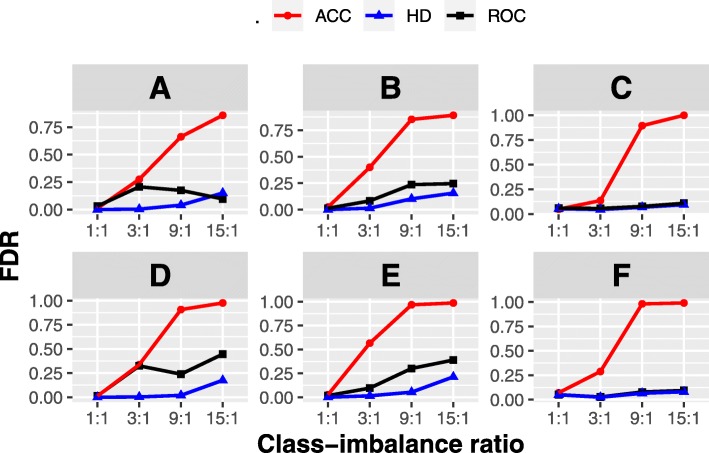
The *FDR* under different class-imbalance ratios. Six subgraphs correspond to six cases shown in Table 1

Figure 3 shows the false discovery rate (*FDR*) derived from ACC, ROC and HD under different class-imbalance ratios. It can be easily seen that HD-based selection outperforms ACC-based and ROC-based selections in terms of *FDR* in most situations. In addition, with the increase of class-imbalance ratio, *FDR* from HD varies very slowly and this trend is much weaker than that from ACC or ROC. This is consistent with the properties of HD.

### Real data study and discussion

#### Data sets and software

The following five gene expression datasets are employed to test the performance of the sssHD algorithm.

**DLBCL dataset**

The DLBCL dataset contains 58 diffuse large B-cell lymphomas (DLBCL) and 19 follicular lymphoma (FL) instances [57]. The original data includes the expression profiles of 7129 genes, and 6285 of them are retained by adopting the data preprocessing method [58]. The class-imbalanced ratio of this set is 3.05, and the selected top *q* predictors are employed to compare our results via several assessment methods, where *q*=1 *to* 10,20,30,40,50,100,200,3142 and 6285.

**SRBCT dataset**

The SRBCT dataset [58, 59] includes 83 instances in total described by 2308 genes in four classes: the Ewing family of tumors (EWS), Burkitt lymphoma (BL), neuroblastoma (NB) and rhabdomyosarcoma (RMS), which have 29, 11, 18, and 25 cases, respectively. To adapt binary classification and follow the partition performed in reference [6], we investigate BL versus the rest, where their sizes are 11 and 72, respectively. The class-imbalanced ratio of this set is 6.55, and the selected top *q* predictors are employed to compare our results via several assessment methods, where the *q*=1 *to* 10,20,30,40,50,100,200,1154 and 2308.

**CAR dataset**

The CAR dataset [60] contains in total 174 instances described by 12533 genes in eleven classes: prostate, bladder/ureter, breast, colorectal, gastroesophagus, kidney, liver, ovary, pancreas, lung adenocarcinomas, and lung squamous cell carcinoma. 9182 of 12533 features are left after doing the data preprocessing [58]. To adapt binary classification and follow the partition performed in the reference [6], we consider class kidney versus the rest, where their sizes are 11 and 163, respectively. Thus the class-imbalanced ratio of this set is 14.82, and the selected top *q* predictors are employed to compared our results via several assessment methods, where *q*=1 *to* 10,20,30,40,50,100,200,4591 and 9182.

**GLIOMA dataset**

The GLIOMA dataset [61] contains in total 50 instances described by 12625 genes in four classes: cancer glioblastomas (CG), non-cancer glioblastomas (NG), cancer oligodendrogliomas (CO) and non-cancer oligodendrogliomas (NO). Among the 50 instances, 14, 14, 7, and 15 cases belong to classes CG, NG, CO and NO, respectively. Among the 12625 genes, 4434^1^ of them remain after data preprocessing [58]. To adapt binary classification and to follow the partition performed in the reference [6], we study the class CO versus the rest, where the numbers of two classes are 7 and 43, respectively. The class-imbalanced ratio of this set is 6.14, and the selected top *q* predictors are employed to compared our results via several assessment methods, where *q*=1 *to* 10,20,30,40,50,100,200,2217 and 4434.

**LUNG dataset**

The LUNG dataset [58] includes five classes, two of them are adenocarcinomas and squamous cell lung carcinomas with sample size of 21 and 20 respectively, and are used in our study. Three of all the 3312 predictors are removed beforehand as they are constant or nearly constant, leaving 3309 predictors after preprocessing. The selected top *q* predictors are utilized to compared our results via several assessment methods, where *q*=1 *to* 10,20,30,40,50,100,200,1654 and 3309. The class ratio of this set is 1.05, and it is employed to mainly exhibit the performance of our proposed algorithm on balanced dataset.

The summary of five data sets is shown in Table 2, and the data is given in the additional files.

**Table 2:** The number of instances, features, majority, and minority as well as the class-imbalanced ratio (CIR) of five datasets used in this study

**Datasets**	**Instances**	**Features**	**Majority**	**Minority**	**CIR**
DLBCL	77	6285	58	19	3.05
SRBCT	83	2308	72	11	6.55
CAR	174	9182	163	11	14.82
GLIOMA	50	4434	43	7	6.14
LUNG	41	3309	21	20	1.05

**Software**

The sssHD algorithm and the related methods or procedures are performed with R language [62], building on packages sparseSVM (https://CRAN.R-project.org/package=sparseSVM), e1071 (https://CRAN.Rproject.org/package=e1071), precrec [48], and ggplot2 [63]. The R code, including the sssHD algorithm and other related procedures, is given in the additional files.

#### Experimental results and discussion

Our algorithm is compared with four methods: Fisher score, Relief, AUCROCfilter and AUCPRCfilter. All methods are performed in two situations: no resampling and resampling with SMOTE [44] over all of the training data sets. SMOTE is an intelligent over-sampling approach, which adds new, artificial minority examples by interpolating between pre-existing minority instances rather than simply duplicating original examples. The minority class is over-sampled by taking each minority class point and introducing synthetic examples along the line segments joining any/all of the *k* minority class nearest neighbors. Depending upon the amount of over-sampling required, the neighbors from the *k* nearest neighbors are randomly chosen.

**Classification results under no resampling**

**Fig. 4:**
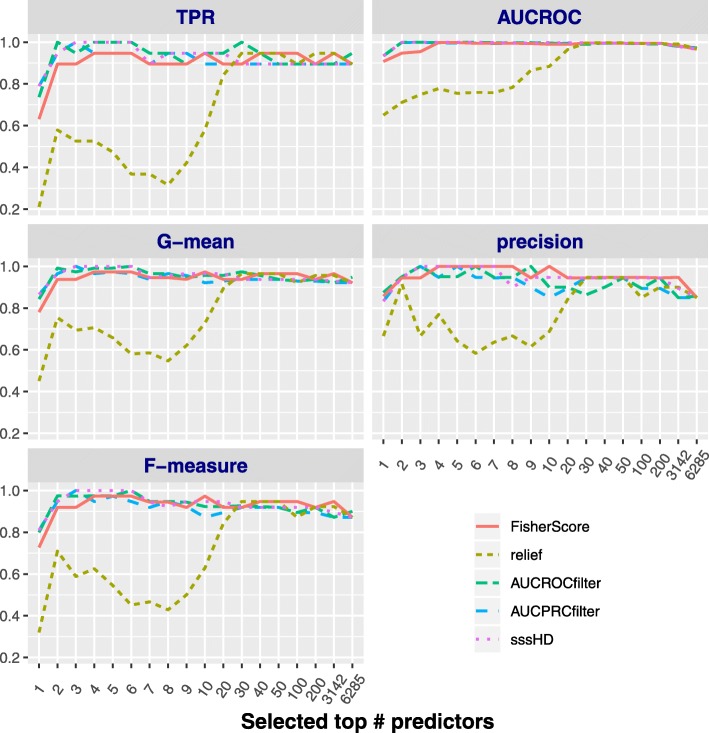
The performance of five methods on DLBCL with no resampling. The five subgraphs report *TPR*, *G*−*mean*, *F*−*measure*, *AUCROC* and *precision*, respectively

**Fig. 5:**
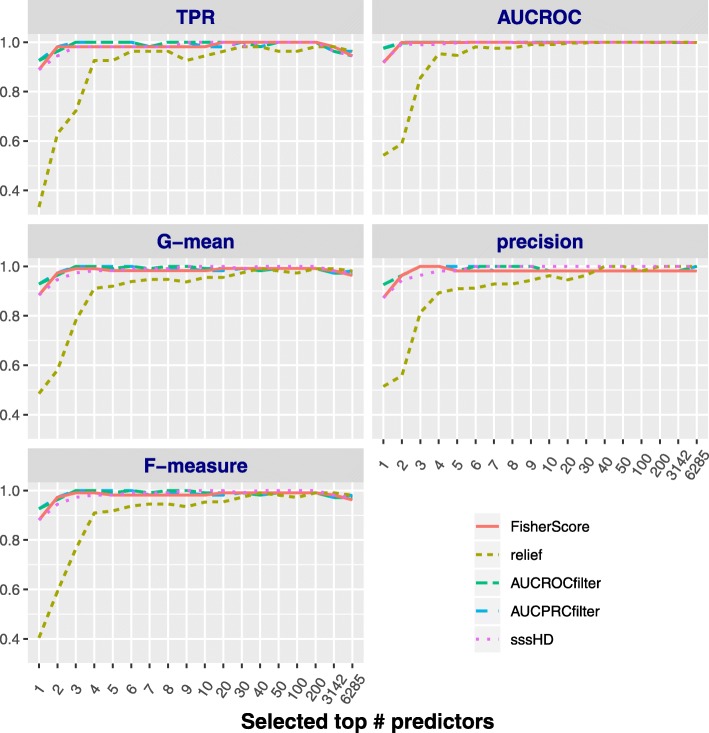
The performance of five methods on DLBCL with SMOTE over-resampling. The five subgraphs report *TPR*, *G*−*mean*, *F*−*measure*, *AUCROC* and *precision*, respectively

In this section, we show the efficacy of the proposed sssHD approach on five gene expression datasets, and compare it with four other filter-based feature selection methods by assessing several performance measurements with no resampling preprocessing. The SVM classifier is employed to finish the classification task. The prediction result is obtained by performing leave-one-out cross validation rather than k-fold cross validation, as it is quite likely that, in the case of class-imbalance, the distribution within each fold varies widely with the uniformly sampling for creating the folds. The results on the first set (DLBCL) are shown in Fig. 4, while the results on last four sets (SRBCT, CAR, GLIOMA and LUNG) are given in the as additional files to save space. Figure 4 includes five subgraphs that evaluate five feature selection methods with *TPR*, *G*−*mean*, *F*−*measure*, *AUCROC* and *precision*, respectively. It can be seen that sssHD gains satisfactory classification performance with just several top-ranked features, regardless of the metric used. The sssHD approach is competitive with other four feature selection methods, especially when the number of top-ranked predictors used is not too large. This finding indicates that the top-ranked features recognized by sssHD actually have the most relevance with the target. Let *q* be the number of top-ranked features that used to firstly reach the maximal value with five metrics. *q* is 3 by sssHD, which is less or equal to the value identified by the other four methods for dataset DLBCL with *G*−*mean*, *F*−*measure*, *AUCROC* and *precision* measurements. Thus sssHD algorithm achieves the best performance with the smallest number of features ranked at the top in the case of no resampling the data. Therefore, sssHD outperforms the other four methods in the original class-imbalanced data. This is also consistent with the property of skew-insensitivity of the Hellinger distance. A similar consequence can be obtained on SRBCT, CAR and GLIOMA datasets; for more details, see SI-Figs. 1, 3, 5 and 7 in the as additional files. In addition, sssHD achieves competitive performance and performs similarly well on balanced dataset LUNG, which further demostrates the skew-insensitivity of HD.

**Classification results with SMOTE resampling**

In this section, we consider the result by implementing re-balancing the data with SMOTE oversampling. The class ratio is approximately 1:1 after doing that. The SVM classifier is still utilized while coupled with stratified 5-fold cross validation due to the balance of the two classes herein. The performance of five methods on the DLBCL data set is shown in Fig. 5. Compared with Figs. 4, 5 shows that the performance with oversampling preprocessing is better than that with no resampling in most cases. Except Relief, the other four methods can obtain satisfactory accuracies, and they have almost no difference. sssHD is less affected by oversampling in comparison with other four methods. This result agrees with two properties of the Hellinger distance: skew-insensitivity and translation-invariant. An interesting discovery is that the optimal number of key features selected under original class-imbalanced data is less than that under SMOTE oversampling. We guess that such an oversampling strategy may lead to over-selection in choosing relevant variables. It also indirectly demostrates that it is necessary to develop feature selection technique designed for original class-imbalanced data rather than re-balanced data. A similar result can be obtained for SRBCT, CAR and GLIOMA datasets by SMOTE preprocessing (LUNG dataset is not performed here due to its balance); for more details, see SI-Figs. 2, 4 and 6 in the as additional files.

It should be pointed out that oversampling with SMOTE is applied before stratified cross validation to keep the class ratios consistent between the training set and the testing set. It is well known that SMOTE is not to simply replicate the original minority instances. In other words, the generated samples are different from the original data and also different from each other. Therefore, the points in testing set are generally not same with those in training set. However, if randomly resampling is used, where new samples are randomly duplicated from the minority class, the instances in testing set are likely to be similar to those in training set consequently leading to enhanced performance.

## Conclusion

In this paper, we proposed a feature selection approach (sssHD) based on the Hellinger distance. Due to the properties of Hellinger distance, the sssHD algorithm is well suited to perform feature selection in class-imbalance learning. We have shown that sssHD can obtain high performance and is extremely competitive against several existing selection methods by means of several assessment measures. sssHD is extremely general as it can be easily extended from at least three aspects: 1) combining with different re-balance samplings such as under-sampling, over-sampling, SMOTE and so on; 2) changing the sparse regularization structure according to the characteristic of the predictor matrix, such as group LASSO [46], if the predictors possess some kind of group structure; and 3) the SVM classifier used in sssHD could be replaced by other classifiers, if necessary, such as discriminant analysis, naive Bayes, logistic regression, random forest, etc.. Therefore, many generalization algorithms can be derived from sssHD. In addition to discovering features that are truly associated with the response, controlling the FDR is also important in performing variable selection [64], so the Hellinger distance coupled with ‘model-X’ knockoffs [65], a useful technique to limit the FDR, would be a feasible choice to recognize true relevant feature and reduce the FDR as much as possible in high-dimensional class-imbalance learning. In addition, the Hellinger distance presents advantages for performing class-imbalanced data, so a worthy attempt may be to directly establish a Hellinger distance feature selection algorithm that does not depend on any classifiers.

## Supplementary information

**Additional file 1** Supporting information is provided in a PDF file, in which the results on SRBCT, CAR and GLIOMA datasets are reposited.

**Additional file 2** Five datasets used in this study are given as a.txt file.

**Additional file 3** The R code for implementing the sssHD algorithm and related calculations is available.

## Data Availability

The supporting information, data as well as the R code for implementing the sssHD algorithm and related procedures are available in the additional files.

## Abbreviations

ACC: accuracy
AUCPRC: area under PRC
AUCROC: area under ROC
C: correctly
CLT: central limit theorem
FDR: false discovery rate
FN: false negative
FP: false positive
HD: Hellinger distance
IC: incorrectly
PRC: precision recall curve
ROC: receiver operating characteristics
sssHD: Hellinger distance based stable sparse selection
SVM: sopport vector machine
TN: true negative
TP: true positive

## Footnotes

1 4433 was given in [58], but a value of 4434 is obtained via the data from [58].
