# Supplementary material for: Hellinger distance-based stable sparse feature selection for high-dimensional class-imbalanced data
Source: BMC Bioinformatics. 2020 Mar 23;21:121. doi: 10.1186/s12859-020-3411-3 (PMC7092448; doi:10.1186/s12859-020-3411-3)
Supplement: Supplementary file 1 — Additional file 1 Supporting information is provided in a PDF file, in which the results on SRBCT, CAR and GLIOMA datasets are reposited. [file 12859_2020_3411_MOESM1_ESM.pdf]

Supporting Information (SI)

Hellinger distance-based stable sparse feature selection for high-dimensional class-imbalanced data

Guang-Hui Fu[1*], Yuan-Jiao Wu[1], Min-Jie Zong[1], Jianxin Pan[2]

Authors affiliations:

[1]School of Science, Kunming University of Science and Technology, Kunming, 650500, China

[2]School of Mathematics, The University of Manchester, Manchester, M13 9PL, UK

Correspondence information

Corresponding author: Guang-Hui Fu.
E-mail address: guanghuifu@kust.edu.cn

The results on SRBCT dataset (SI-Fig. 1 to 2)

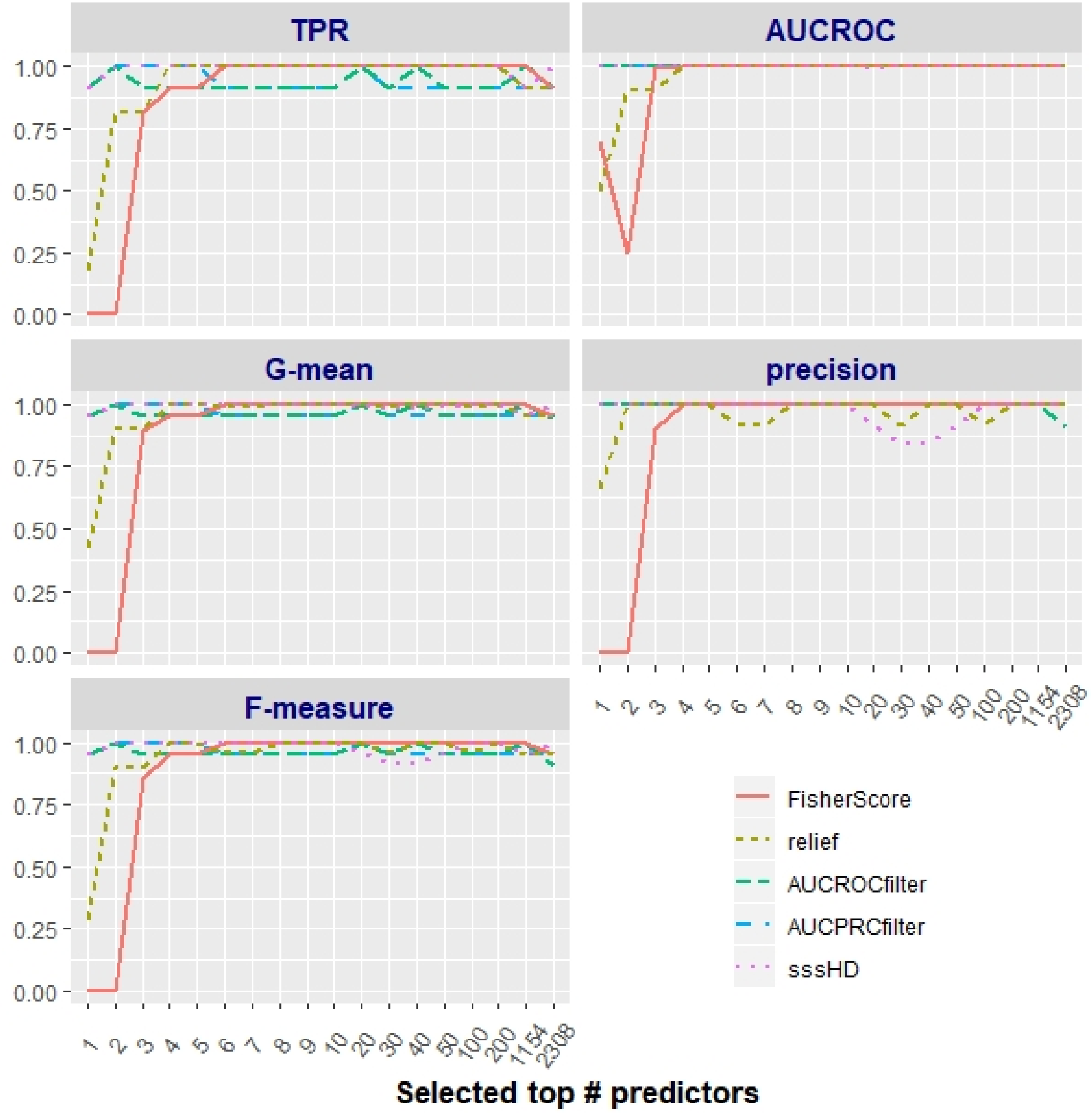

SI-Fig. 1 The performance of five methods on SRBCT with no resampling.

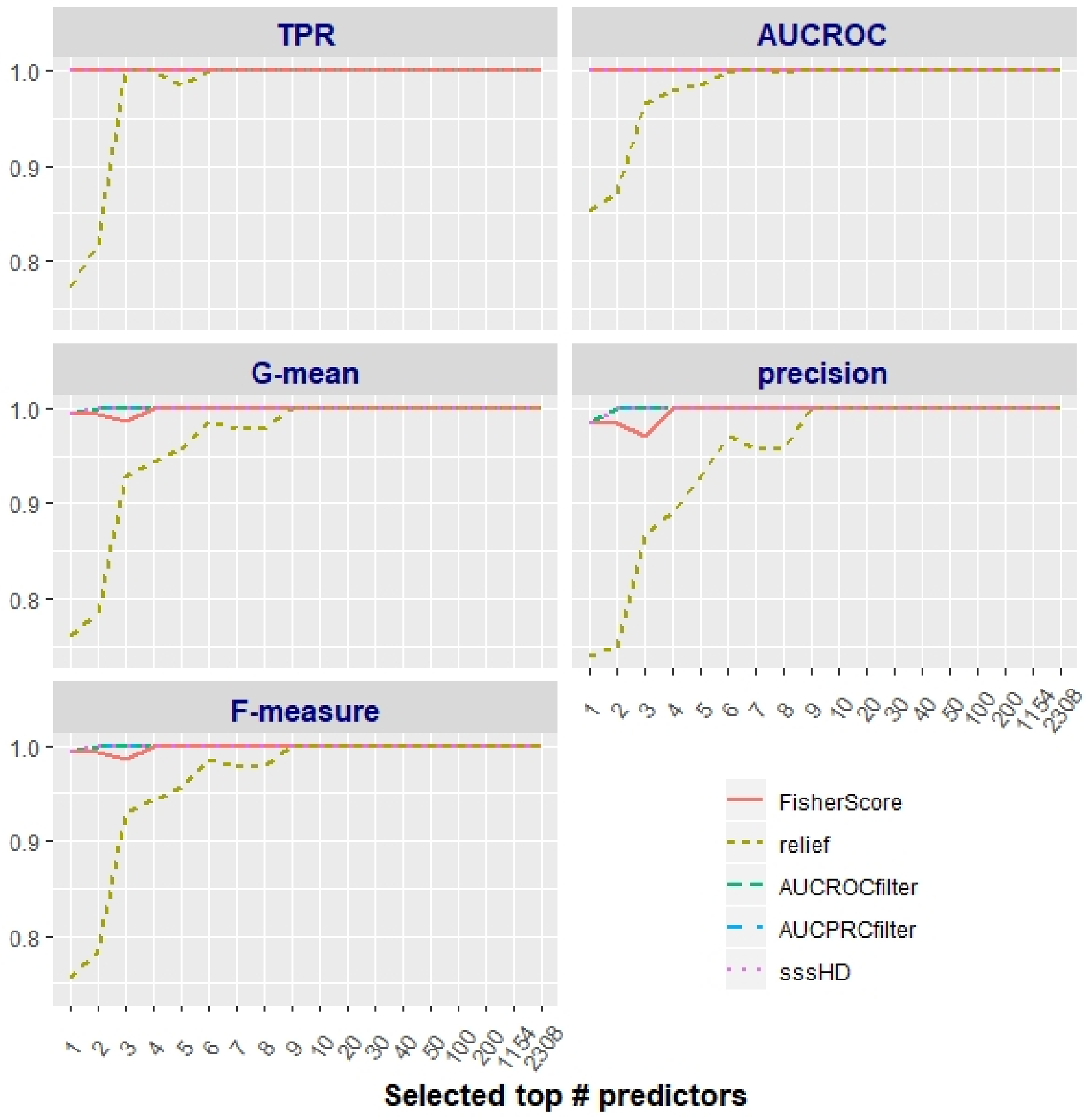

SI-Fig. 2 The performance of five methods on SRBCT with SMOTE over-resampling.

The results on CAR dataset (SI-Fig. 3 to 4)

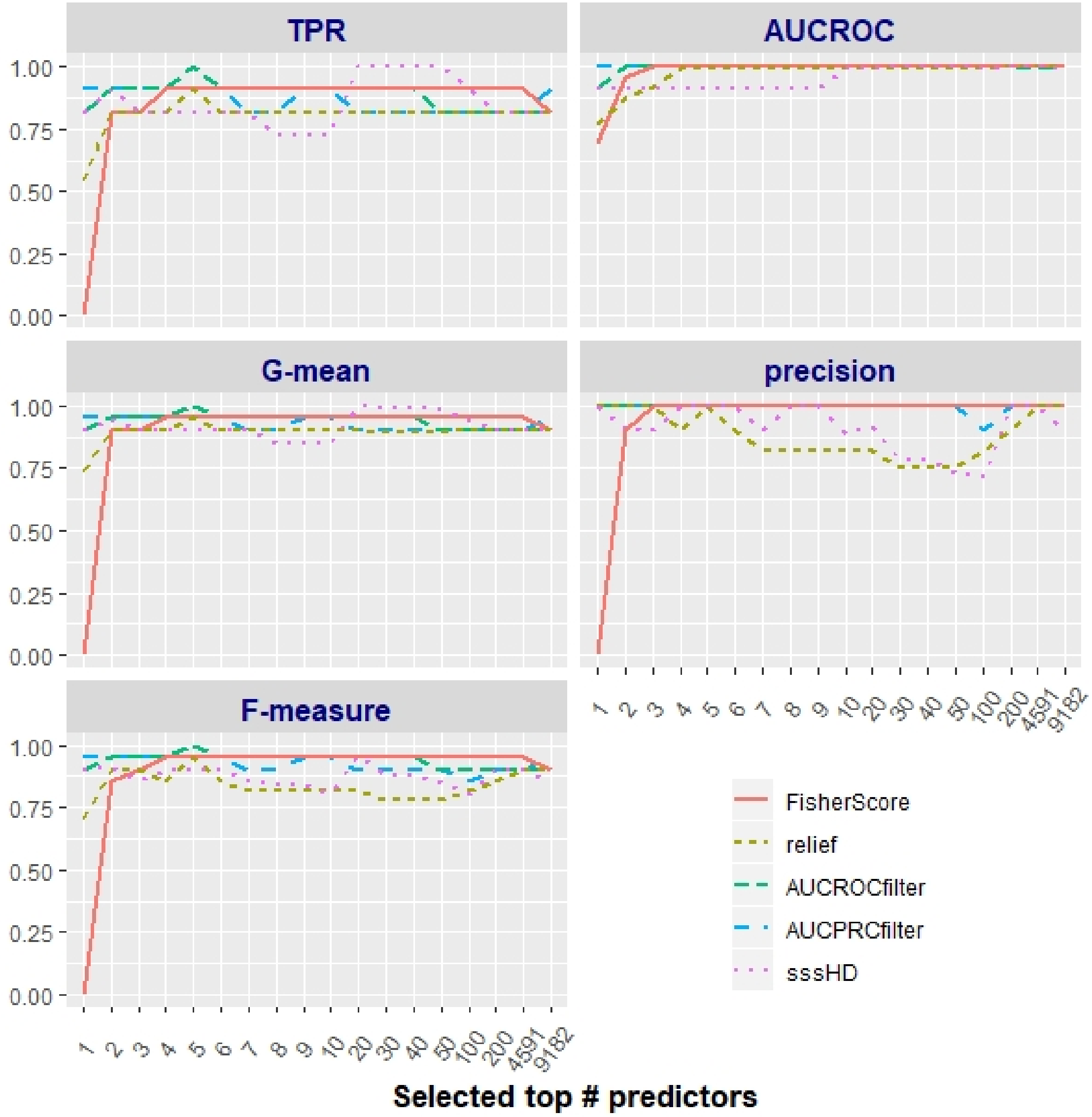

SI-Fig. 3 The performance of five methods on CAR with no resampling.

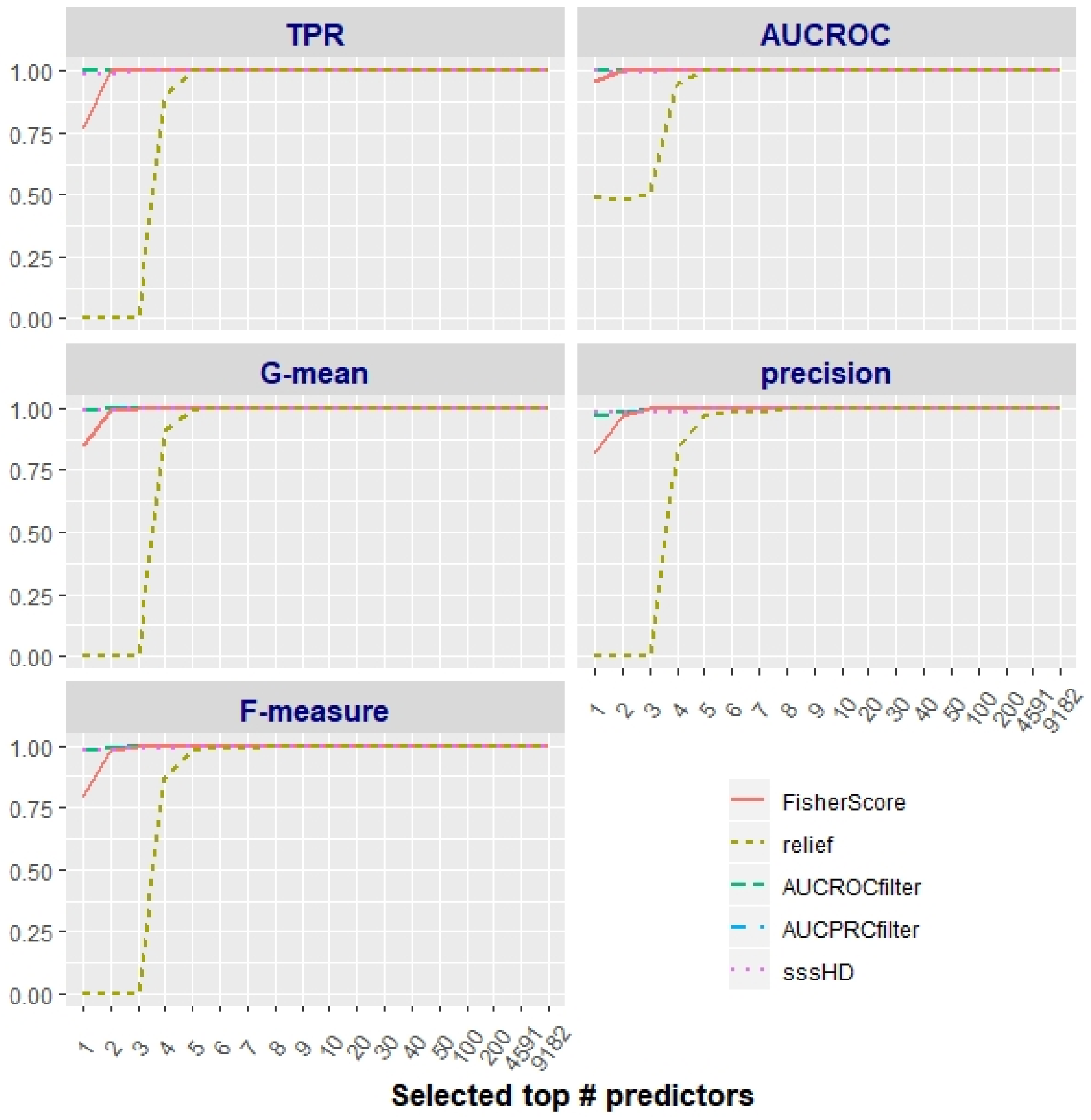

SI-Fig. 4 The performance of five methods on CAR with SMOTE over-resampling.

The results on GLIOMA dataset (SI-Fig. 5 to 6)

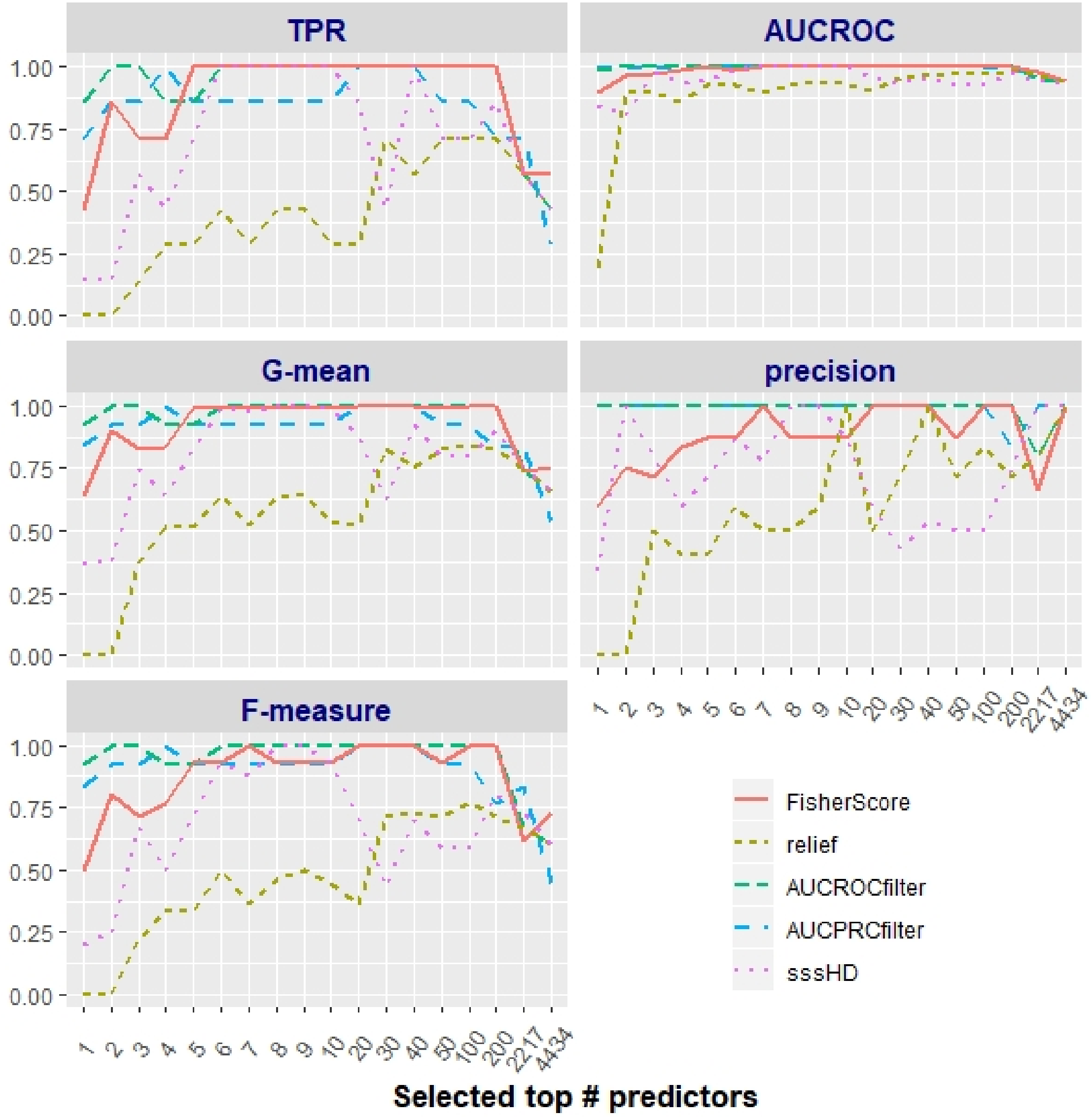

SI-Fig. 5 The performance of five methods on GLIOMA with no resampling.

The results on GLIOMA dataset (SI-Fig. 7)

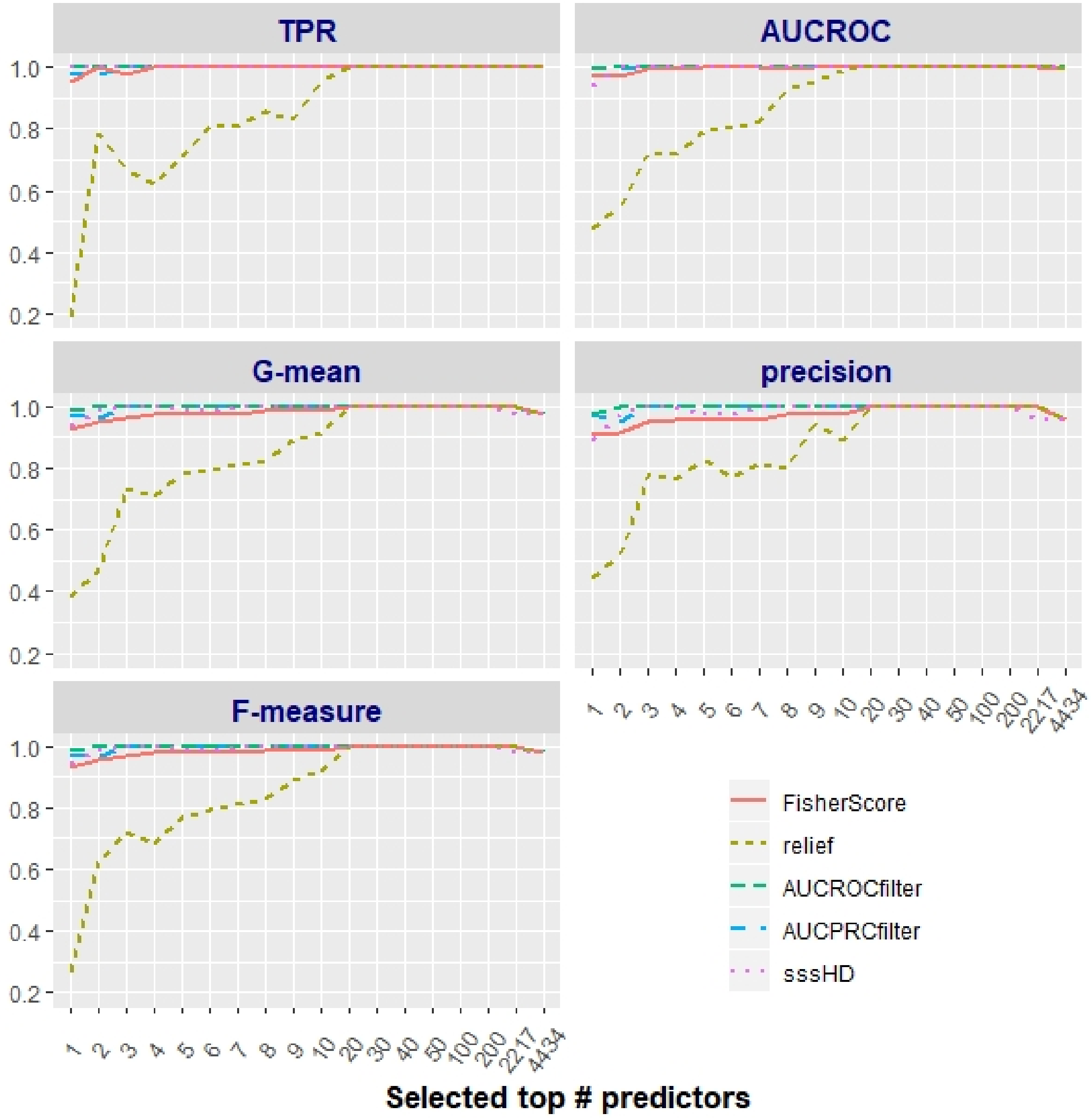

SI-Fig. 7 The performance of five methods on GLIOMA with SMOTE over-resampling.

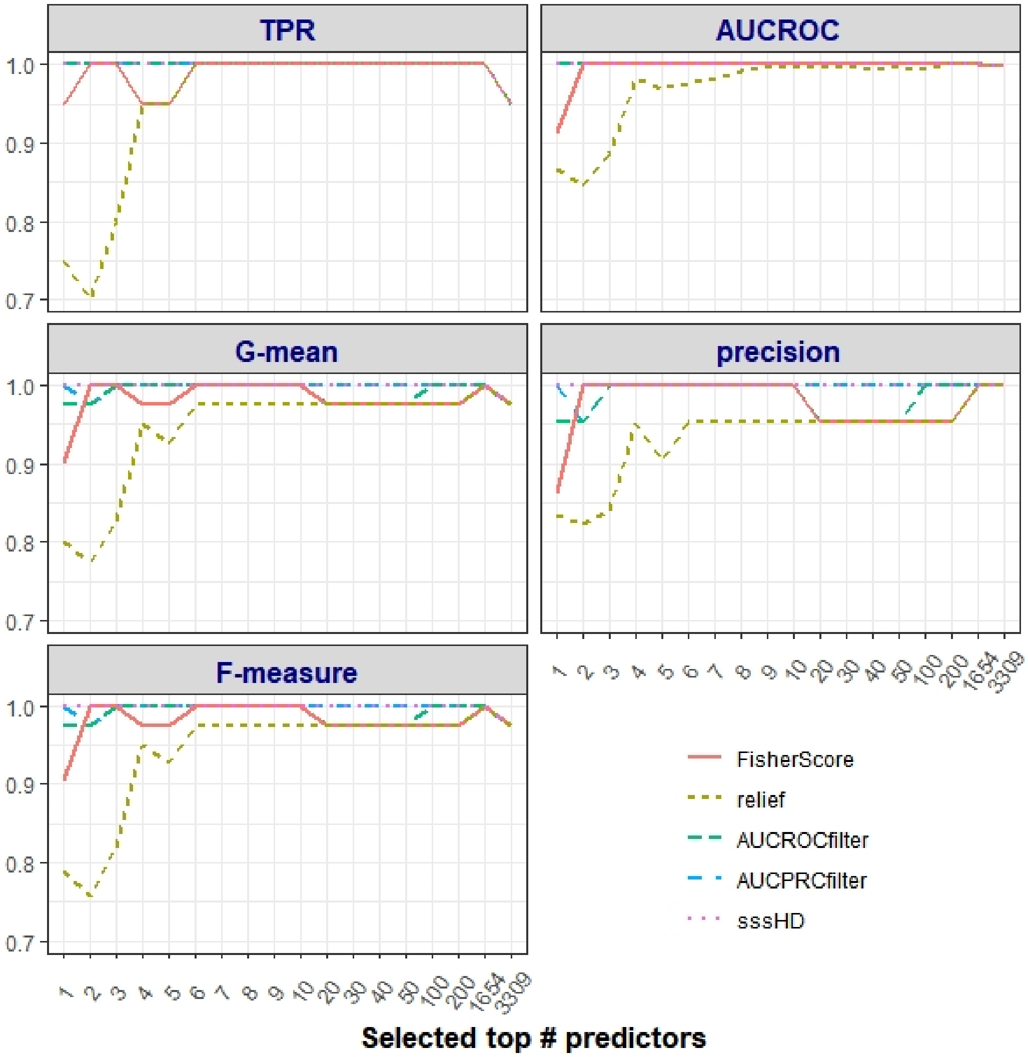

SI-Fig. 7 The performance of five methods on LUNG with no resampling.
